# Scheimpflug Imaging Parameters Associated with Tear Mediators and Bronchial Asthma in Keratoconus

**DOI:** 10.1155/2016/9392640

**Published:** 2016-01-12

**Authors:** Dorottya Pásztor, Bence Lajos Kolozsvári, Adrienne Csutak, András Berta, Ziad Hassan, Beáta Andrea Kettesy, Péter Gogolák, Mariann Fodor

**Affiliations:** ^1^Department of Ophthalmology, University of Debrecen, Debrecen 4012, Hungary; ^2^Orbident Refractive Surgery and Medical Center, Debrecen 4012, Hungary; ^3^Department of Immunology, University of Debrecen, Debrecen 4012, Hungary

## Abstract

*Purpose.* To determine associations between mediators in tears in the whole spectrum of keratoconus (KC); to explore connections between mediators and Scheimpflug parameters; to examine correlations between Scheimpflug parameters and bronchial asthma.* Methods.* Tear samples were collected from 69 patients and 19 controls. Concentrations of mediators—IL-6, -10; CXCL8, CCL5; MMP-9, -13; TIMP-1; t-PA, PAI-1—were measured by Cytometric Bead Array. Measured Pentacam parameters include keratometry values (*K*_1_, *K*_2_, *K*_max_), corneal thickness (Pachy Pupil, Apex, Min), and elevations and indices (including Belin-Ambrósio deviation (BAD-D)).* Results.* A number of significant positive associations were observed between pairs of mediator concentrations. Significant positive correlations were found between BAD-D and CXCL8/MMP-9 and *K*_2_ and MMP-9. Significant negative associations were explored between Pachy Min and CXCL8/t-PA. Significant associations were found between pairs of mediators (IL-6 and CXCL8; CCL5 and CXCL8/MMP-9; TIMP-1 and MMP-9/-13/t-PA; t-PA and CXCL8/CCL5/PAI-1) and the severity of KC. Significant positive correlation between asthma and the severity of KC was explored.* Conclusion.* Cooperation of different mediators in tears all taking part in the complex pathomechanism of keratoconus was revealed. Our research verifies that inflammation plays a crucial role in the pathogenesis of KC. Additionally this study confirms the effect of bronchial asthma on keratoconus.

## 1. Introduction

Keratoconus (KC) is usually a bilateral, progressive ectatic corneal disorder, usually appearing in puberty [1–3]. The corneal stroma becomes thinner and protrudes, causing the typical conical shape that leads to irregular astigmatism, myopia, and the decrease of visual acuity [1–3]. The apex of the protrusion can most commonly be found in inferotemporal orientation from the center of the cornea [1, 2]. The incidence of KC is around 1 : 2,000 in the general population [2]. The etiology of the disease is not yet known in detail [1–4].

Classically, KC is considered to be a noninflammatory disease [1]. However, recent studies have suggested that inflammatory factors play a key role in the pathomechanism of the disorder [4]. Elevated levels of interleukin- (IL-) 1b, IL-4, IL-5, IL-6, IL-8, and IL-17; tumor necrosis factor (TNF)-*α*, -*β*; interferon- (IFN-) *γ*; matrix metalloproteinase- (MMP-) 1, MMP-3, MMP-7, MMP-9, and MMP-13; Cathepsin B; and Lipocalin-1 have been found in the tears of patients with keratoconus [4–11]. Decreased levels of IL-4, IL-10, IL-12, and IL-13; TNF-*α*; IFN-*γ*; Chemokine (C-C motif) ligand 5 (CCL5/RANTES, regulated on activation, normal T cell expressed and secreted); lipophilin C; lipophilin A; lactoferrin; *α*-fibrinogen; zinc-*α*2-glycoprotein (ZAG); immunoglobulin A (IgA); immunoglobulin *κ*-chain (IGKC); polymeric immunoglobuline receptor (PIGR); phospholipase A2; cystatin S; cystatin SN and cystatin SA have also been found in the tear fluid [4, 8, 10–14]. Kenney et al. have found a decreased level of tissue inhibitor of metalloproteinase-1 (TIMP-1) in KC corneas, compared to normal corneas [15].

In addition, an association between KC and bronchial asthma has been identified almost 50 years earlier [16]. Several studies have demonstrated a strong association between KC, asthma, and other immune disorders, pointing to the crucial role of the immune system in the pathogenesis of keratoconus [17, 18].

In the past few decades, slit-imaging technologies provided further improvement in corneal imaging. Nowadays, we can measure not only the front but also the back surface of the cornea with pachymetric mapping and can typify corneal architecture in three dimensions. Furthermore, Ambrósio et al. established numerous indices to improve the screening of the ectasia [19, 20].

There are only a few preliminary studies examining the association between mediators (mainly cytokines) in the tear fluid and the severity of keratoconus. Lema and Durán analyzed 28 eyes [5], Jun et al. checked 18 patients [13], and Kolozsvári et al. studied only 14 keratoconic eyes [21]. In a recent study, Shetty et al. [22] examined the association between MMP-9, IL-6, TNF-*α*, and different stages of KC. The crucial limitations of these studies [5, 13, 21, 22] are the small number of patients, or the few examined mediators, or the lack of subclinical cases.

In the present study, our goals were to determine associations between the different types of mediators in tear fluid—IL-6, IL-10, chemokine (C-X-C motif) ligand 8 (CXCL8)/IL-8, CCL5/RANTES, MMP-9, MMP-13, TIMP-1, tissue plasminogen activator (t-PA), and plasminogen activator inhibitor (PAI-1)—in the whole spectrum of keratoconic eyes (suspect, subclinical, and manifest cases of keratoconus) and normal eyes. An additional goal was to explore associations between these mediators and the Scheimpflug parameters which characterize the severity of keratoconus. Our aim was also to examine the relationship between the Scheimpflug imaging parameters and bronchial asthma in keratoconus.

## 2. Patients and Methods

### 2.1. Subjects and Clinical Examinations

In this prospective study, patients with keratoconus and normal subjects were recruited from the Outpatient Unit, Department of Ophthalmology, Faculty of Medicine, University of Debrecen, Hungary.

We examined patients with keratoconus at all stages (severe, moderate, and mild KC, subclinical KC or forme fruste KC) and normal, control patients. We have categorized the participants based on the clinical stage of keratoconus, but group allocation was not a factor in the analysis. An eye was diagnosed as having keratoconus where it had one or a combination of the following clinical signs: central or paracentral stromal thinning of the cornea, conical protrusion, Fleischer's ring, Vogt's striae by slit-lamp examination, and topographic changes [23]. The stages of KC were divided between mild, if the steepest keratometric reading (*K*_2_) was <45 diopters (D); moderate, when *K*_2_ was between 45 and 52 D; and severe if *K*_2_ was >52 D [13, 24]. At present, there are no specific or universally accepted criteria categorizing an eye as having subclinical KC or forme fruste KC (FFKC). The criteria for diagnosing subclinical KC or FFKC were defined as one or a combination of the following clinical signs: if it was the fellow eye of a patient with keratoconus and had a normal cornea on slit-lamp examination (in several cases, keratoplasty or corneal cross-linking was performed on the keratoconic eye), or using a Pentacam, the BAD-D (Belin-Ambrósio deviation index) [20], ART Max (Maximum Ambrósio's Relational Thickness) [19], and PPI Ave (Average Pachymetric Progression Index) [19]; or where the back elevation values at the thinnest location (B.Ele.Th.) were not in the normal range of the Pentacam; or where the criteria of KC did not apply but the maximum keratometric reading of the front surface (*K*_max_ Front) was more than 47 D.

We included a randomly selected eye from each participant, although both eyes were examined. Eye selection was based on generating random values using Microsoft Excel set to produce numeric indicators with equal probabilities for either eye. Altogether, 69 patients (mean (SD) age 30.7 (10.3), range 13–68 years) with the following diagnoses—severe KC: 25, moderate KC: 21, mild KC: 5 and subclinical KC: 18—and 19 normal controls (mean (SD) age 31.7 (11.5), range 18–67 years) were enrolled in the study. The 69 eyes of the patients represented the whole spectrum of the abnormal, ectatic, and keratoconic corneas, although no such group allocation was made.

The exclusion criteria included the existence of active inflammatory or infective systemic or ocular disease, eye rubbing, and current treatment with systemic or local anti-inflammatory drugs. Those eyes that were affected by previous ocular surgery or trauma, as well as those patients who were pregnant or lactating during the course of the study, were also excluded. Written informed consent from each participant, as well as permission from the University of Debrecen Institutional Ethics Committee, was obtained prior to enrollment. The tenets of the Declaration of Helsinki were followed in all procedures during the study.

Both eyes of each participant underwent ophthalmological evaluation, including clinical history (especially bronchial asthma and contact lens usage), automated kerato-refractometry (KR-8900; Topcon Co, Tokyo, Japan), uncorrected and corrected distance visual acuity determinations, slit-lamp biomicroscopy (under low illumination to avoid reflex tearing), Rotating Scheimpflug tomography (Pentacam HR, Oculus Optikgeräte GmbH, Wetzlar, Germany), and nonstimulated tear sample collection with glass capillaries.

### 2.2. Pentacam Measurements

All eyes were examined with a Pentacam HR (software version 1.16r26 and 1.17r139) without the application of any eye drops. Three sequential scans were taken in each eye by the same trained examiner. In short, the patients were asked to keep both eyes open and fixate on the target, in the center of the blue fixation beam. The examiner with the joystick then focuses and obtains a correct alignment with the corneal apex and a perfect focus, and the instrument automatically takes 25 Scheimpflug images within two seconds. The quality of images was checked under the quality specification (QS) window and only the correct measurements (“QS” reads “OK”) were accepted; if the comments were marked yellow or red, the examination was repeated [25, 26].

The following parameters were exported to Microsoft Excel (Microsoft Corp, Redmond, Washington): Holladay equivalent keratometry values in the flat (*K*_1_) and steep (*K*_2_) meridian; maximal keratometry of the front surface (*K*_max_ Front); corneal astigmatism (Astig); corneal thickness at the pupil's center (Pachy Pupil), at the apex (Pachy Apex) and at the thinnest point of the cornea (Pachy Min); Index of Surface Variation (ISV); Index of Vertical Asymmetry (IVA); Keratoconus Index (KI); Central Keratoconus Index (CKI); Index of Height Asymmetry (IHA); Index of Height Decentration (IHD); front and back elevation at the thinnest location (F.Ele.Th. and B.Ele.Th.); minimal, maximal, and average pachymetric progression indices (PPI Min, PPI Max, and PPI Ave); Ambrósio's Relational Thickness (ART); and Belin-Ambrósio deviation index (BAD-D).

### 2.3. Tear Collection and Analysis

Nontraumatic tear collection was carried out using sterile thin glass microcapillary tubes from the inferior meniscus, without anesthetic drops or stimulation. Tears were collected for two minutes and then promptly transferred to Eppendorf tubes and frozen at −80°C without centrifugation, within 15 minutes of collection. The samples were stored until they were analyzed. In all cases, the total volume of collected tear samples was registered. We calculated tear volumes from the length of the tear column in the tube.

In tear samples, the concentrations of IL-6, IL-10, CXCL8/IL-8, CCL5/RANTES, MMP-9, MMP-13, TIMP-1, t-PA, and PAI-1 were measured using the Cytometric Bead Array method. Combined FlowCytomix Simplex Kits were used with the suitable FlowCytomix Basic Kit, with minor modifications to the manufacturer's orders (eBioscience, Bender Med Systems GmbH, Vienna, Austria) [27]. Briefly stated, 12.5 *µ*L of tear samples (diluted samples, if necessary) or serial dilutions of mixed standard cytokines were added to a 12.5 *µ*L suspension of fluorescent cytokine capture beads in multiwell filter microplates. Added to the wells were 12.5 microliters of biotin-conjugated anti-cytokine antibodies, after which the plates were incubated for two hours on a microplate shaker. The wells were emptied and washed with a vacuum filtration manifold. Phycoerythrin-conjugated streptavidin was added to the plate wells, followed by an additional incubation period of one hour and washed as described before. A 150 *µ*L assay buffer was applied to the wells, after which multiparametric data acquisition was executed using a FACS Array cytometer (BD Biosciences Immunocytometry Systems, San Jose, CA). The data were analyzed with the FlowCytomix Pro 2.3 software (eBioscience). Additional serial dilutions of the standard were applied to achieve better sensitivity and modified standard curves were thus generated in the analysis. The subsequent detection limits were as follows: IL-6: 1.2 pg/mL; IL-10: 1.9 pg/mL; CXCL8 (IL-8): 0.5 pg/mL; CCL5 (RANTES): 25 pg/mL; MMP-9: 95 pg/mL; MMP-13: 50 pg/mL; TIMP-1: 28 pg/mL; t-PA: 4.8 pg/mL; and PAI-1: 13.5 pg/mL.

### 2.4. Statistical Analysis

We categorized the participants for descriptive purposes based on clinical severity (group allocation was not used as a variable in the analysis). Mediator concentration variables were inspected for the distribution shape and natural log transformed (for t-PA and PAI-1, square root transformed) to improve normality. Similarly, Pentacam parameters were subjected to one of these transformations if that improves distributional symmetry.

Pentacam parameters were unified in a composite index referred to as the Standardized Pentacam Score. This was calculated by centering, standardizing, and direction correcting the source variables (so that the higher values invariably represent more severe pathologies), running a principal component analysis and deriving the first principal component.

Associations between all possible pairs of mediator levels, as well as those between mediators in the tear fluid and Scheimpflug parameters, were evaluated using simple linear regression, including a quadratic term by a curvature in the relationship, if required. Associations between pairs of mediators and the Standardized Pentacam Score were assessed using multiple linear regressions adjusted for age, the presence of asthma, and contact lens usage. Mediator variables were used in linear and quadratic forms, and interactions between those terms were also included in order to accommodate the model to curvatures in the outcome space. Relationships were expressed as the overall significance of the mediator pair effect and also as additive differences in the Standardized Pentacam Score at sample-covered locations, defined by mediator pair concentrations versus an arbitrary reference point.

## 3. Results

### 3.1. Associations between Mediator Levels in the Tear Fluid

A number of significant positive associations were observed between pairs of mediator concentrations in tear fluid collected from KC patients and subjects with normal eyes, as shown in Table 1.

### 3.2. Associations between Mediators in the Tear Fluid and Scheimpflug Parameters

Significant positive associations were found between BAD-D and CXCL8, BAD-D and MMP-9, and *K*_2_ and MMP-9. Significant negative associations were found between Pachy Min and CXCL8 and Pachy Min and t-PA (Table 2).

### 3.3. Associations between Pairs of Mediators and the Standardized Pentacam Score (A Composite Parameter Calculated from Pentacam Readings)

A significant association was found between the TIMP-1 concentration, the MMP-9 concentration, and the Standardized Pentacam Score: the combination of high TIMP-1 and low MMP-13 levels was characterized by a low score, while high levels of both mediators—as well as low TIMP-1 concentrations coupled with moderate MMP-9 levels—were associated with an elevated score (Table 3).

Significant associations were also found between the concentrations of a number of other pairs of mediators and the Standardized Pentacam Score as shown in Table 4.

### 3.4. The Effect of Bronchial Asthma on Standardized Pentacam Score

There was a history of asthma in five patients (7.25%) and one of contact lens usage in 15 patients (21.74%). A strong, significant positive association between asthma and the Standardized Pentacam Score was found by linear regression adjusted for age and contact lens usage. Asthmatic patients' scores were, on average, an estimated 5.7 units (95% CI: 2.0 to 9.4, *p* = 0.003)—or 1.32 standard deviations—higher than those of subjects without the condition.

## 4. Discussion

To the best of our knowledge, this is the first study that aimed to reveal associations between pairs of mediators and the severity of keratoconus, evaluated using a Pentacam. Despite the intensive clinical and biochemical investigations, the pathogenesis of keratoconus is not yet known in detail. Classically, keratoconus was considered a noninflammatory disease [1]; however, recently published articles have suggested that inflammation is involved in the pathogenesis of KC [4, 7–14]. In the tear fluid of keratoconic patients, elevated levels of IL-6, TNF-*α*, and MMP-9 were detected, and IL-6 and TNF-*α* levels were also elevated in subclinical cases [5, 6]. Jun et al. found high level of IL-6 and low levels of IL-12, TNF-*α*, IFN-*γ*, IL-4, IL-13, and CCL5 in the tear fluid of KC patients [13]. Partly in line with these results, increased tear levels of MMP-1, MMP-3, MMP-7, MMP-9, and MMP-13; IL-4, IL-5, IL-6, and IL-8, and TNF-*α*, -*β* were found in keratoconus [9]. In keratoconic corneas, a decrease in TIMP-1 mRNA was reported [15]. Only few studies investigated the associations between a range of cytokines in the tear fluid and the severity of keratoconus. The limitations of these reports are the small number of patients, or the few examined mediators, or the lack of subclinical cases [5, 13, 21, 22].

In the current study, we have determined the associations between nine mediators (IL-6, IL-10, CXCL8/IL-8, CCL5/RANTES, MMP-9, MMP-13, TIMP-1, t-PA, and PAI-1) in the tear fluid of keratoconic patients covering the whole spectrum of the disease (from subclinical to manifest keratoconus). In accordance with earlier studies, we proved that different mediators—including cytokines, chemokines, enzymes, and inhibitors—in the tear fluid cooperate and take part in a complex immunological network [4, 5, 7–14, 21]. Significant associations were explored between the concentrations of different mediators (between IL-6 and CXCL8; between CCL5, CXCL8, and MMP-9; between TIMP-1, MMP-9, MMP-13, and t-PA; and between t-PA, CXCL8, CCL5, and PAI-1) and the Standardized Pentacam Score, which is a composite index statistically unifying all Pentacam parameters. As far as we know, there are no studies evaluating the association between different tear mediators and Pachy Min and also BAD-D, which was designed to present comprehensive data based on anterior and posterior corneal elevation and a pachymetric evaluation. Our results support the linkage between the complex network of the various mediators and the comprehensive Pentacam indices. Based on our results, inflammation not only seems to be involved in the pathogenesis of keratoconus, but also plays a crucial role in the pathological corneal processes from the initial stage to the final one. We have found only a few significant associations between the single mediators and Pentacam indices, highlighting the fact that mediators, including cytokines, take part in a complex cascade. The examined mediators overlap, neutralize and enhance the effects of one another. It is in line with evidence showing that various multitargeted mediators in the serum collaborate in different diseases, suggesting that examination of only one or a few mediators is not enough to explore complex immunopathological processes [13, 28, 29].

MMPs are zinc-dependent endopeptidases, which participate in degrading and remodeling the extracellular matrix, thereby maintaining its integrity during normal conditions. Nevertheless, under pathological conditions, MMPs can support tissue destruction and other inflammatory reactions. TIMP-1 is the inhibitor of MMPs, which prevents pro-MMP activation and, furthermore, presents antiapoptotic properties [15, 30, 31]. MMP-13 was categorically reported in keratoconus, suggesting that it plays a role in intra- and extracellular pathological collagen destruction [32]. Additionally, proMMP-13 activation is partially inhibited by TIMP-1 [33]. In line with these studies, we have found significant associations between TIMP-1 concentration, MMP-13 concentration, and the Standardized Pentacam Score. The combination of high TIMP-1 and low MMP-13 levels was characterized by a low score, while high levels of both mediators—as well as low TIMP-1 concentrations coupled with moderate MMP-9 levels—were associated with elevated scores. Additionally, significant positive associations were found between MMP-9 and BAD-D, and also with *K*_2_.

Based on our study and others, the collagenolytic milieu of the human cornea seems to be more complex than expected. Further studies are required to understand the exact mechanisms of collagenases and inhibitors.

An active form of t-PA converts plasminogen to plasmin and can also degrade several components of the extracellular matrix, triggering the activation of the MMP pathway. Numerous interactions have been observed between the fibrinolytic and MMP systems taking part in proteolytic activation. Plasminogen activators are partially regulated by PAIs, inhibiting this cascade system and, therefore, influencing KC progression [34]. Different growth factors and cytokines induce the PAI-1 gene and inhibit the activity of the t-PA enzyme. In addition, PAs could affect the proteolytic inactivation of growth factors [35]. Significant associations between the severity of keratoconus and pairs of t-PA/TIMP-1 and t-PA/PAI were detected in our study. In addition to this observation, significant negative associations were found between the corneal thickness at the thinnest point of the cornea and t-PA.

Apart from the elements of the proteolytic and fibrinolytic systems, we have examined different cytokines, such as the proinflammatory IL-6 and chemokine CXCL8/IL-8 and the anti-inflammatory Th2 cytokine IL-10 [13]; we found that all of these cytokines cooperate with each other and play a crucial role in the pathogenesis of KC. The significant positive association between BAD-D and CXCL8 and the significant negative correlation between Pachy Min and CXCL8 highlight the roles of the chemokines.

Bronchial asthma is a chronic airway inflammatory disease. The infiltration of eosinophils, mastocytes, and T lymphocytes and the release of several inflammatory mediators play an important role in asthma's pathogenesis [36, 37]. Different proteins are altered in the serum of these patients, such as IgE; IL-1, IL-4, IL-6, IL-8, and IL-13; CCL5; TNF-*α*; MMP-9; TIMP-1; t-PA; and serum Angiopoietin-1 [36, 38–43]. Due to an increase in the number of known mediators, additional anti-inflammatory options are becoming available in the therapy of asthma. The connection between asthma and other atopic diseases with keratoconus was first published almost 50 years ago and has been confirmed several times since then [16–18]. We found a strong significant positive association between asthma and the severity of keratoconus, meaning that asthmatic patients have 5.7 higher score than nonasthmatic subjects. Based on our study, bronchial asthma has an impact on the severity of keratoconus. This result albeit was a secondary outcome of our study and confirms the previous hypothesis related to the connection between KC and asthma. Further larger randomized studies are required to verify this strong correlation.

The strengths of our study are the large number of participants (88 subjects) and the consideration of a wide range of tear mediators that could be associated with Pentacam parameters. Our study has limitations, such as the lack of examination of enzyme activities, as well as the fact that we did not examine the progression of keratoconus and the effect of asthma medication. In addition, this study does not exclude the role of other inflammatory molecules in the pathophysiology of KC. It would be interesting to measure more and different types of mediators, but this remains to be determined in subsequent studies. However, several new correlations can be revealed from our results that can be the basis for further study of this topic.

## 5. Conclusion

Keratoconus has a complex pathomechanism, in which many different cytokines, chemokines, enzymes, and inhibitors are involved. This study reveals the cooperation of the different mediators in tear fluid all taking part in this complex immunological network. As far as we know, this is the first study to reveal associations between tear mediators and BAD-D or Pachy Min. Our study confirms that inflammation is involved in the pathogenesis of keratoconus. As a next step, the precise role of these mediators needs to be defined, as well as examination of the progression of KC and exploration of other mediators' functions. These studies might then serve as a platform for finding targets for local inhibition of pathological corneal thinning, or eventual treatment. In addition, our study confirms the effect of bronchial asthma on keratoconus. Further prospective studies are required to examine the effect of the systemic treatment of asthma on the pathomechanism of keratoconus.

## Figures and Tables

**Table 1:** Significant positive associations between the concentrations of the different tear mediators.

	IL-6 (pg/mL)	IL-10 (pg/mL)	CXCL8 (pg/mL)	CCL5 (pg/mL)	MMP-9 (ng/mL)	MMP-13 (ng/mL)	TIMP-1 (ng/mL)	t-PA (pg/mL)	PAI-1 (ng/mL)
IL-6 (pg/mL)		*p* < 0.0001	*p* < *0.0001* ^*∗*^	*p* < 0.0001	*p* = 0.0203	*p* < *0.0001* ^*∗*^	*p* < *0.0001* ^*∗*^	*p* < *0.0001* ^*∗*^	*p* < 0.0001
IL-10 (pg/mL)			*p* < 0.0001	*p* < 0.0001		*p* < 0.0001		*p* < 0.0001	*p* < *0.0001* ^*∗*^
CXCL8 (pg/mL)				*p* < 0.0001	*p* < 0.0001	*p* < 0.0001	*p* < 0.0001	*p* < *0.0001* ^*∗*^	*p* < 0.0001
CCL5 (pg/mL)						*p* < 0.0001		*p* < *0.0001* ^*∗*^	*p* < 0.0001
MMP-9 (ng/mL)							*p* < 0.0001	*p* < 0.0001	
MMP-13 (ng/mL)							*p* = *0.0001* ^*∗*^	*p* < 0.0001	*p* < *0.0001* ^*∗*^
TIMP-1 (ng/mL)								*p* < *0.0001* ^*∗*^	*p* < *0.0001* ^*∗*^
t-PA (pg/mL)									*p* < *0.0001* ^*∗*^
PAI-1 (ng/mL)									

IL: interleukin; CXCL8: chemokine (C-X-C motif) ligand 8 = IL-8; CCL5: chemokine (C-C motif) ligand 5 = RANTES: regulated on activation, normal T cell expressed and secreted; MMP: matrix metalloproteinase; TIMP-1: tissue inhibitor of metalloproteinase-1; t-PA: tissue plasminogen activator; PAI-1: plasminogen activator inhibitor (shape of relationship: linear, *quadratic* ^*∗*^).

**Table 2:** Significant associations between tear mediators and Pentacam indices.

	CXCL8 (pg/mL)	MMP-9 (ng/mL)	t-PA (pg/mL)
BAD-D	*p* = 0.020	*p* = 0.005	
*K*_2_ F		*p* = 0.031	
Pachy Min	**p** = 0.027		**p** = 0.023

BAD-D: Belin-Ambrósio deviation index; KI: keratoconus Index; *K*_2_ F: Holladay equivalent keratometry value in the steep meridian on the front surface; Pachy Min: corneal thickness at the thinnest point of the cornea; CXCL8: chemokine (C-X-C motif) ligand 8 = IL-8: interleukin-8; MMP-9: matrix metalloproteinase-9; t-PA: tissue plasminogen activator (bold values represent negative correlations).

**Table 3:** Additive differences in the Standardized Pentacam Score at various locations defined by TIMP-1 concentration (ng/mL) and MMP-13 concentration (ng/mL) versus the indicated reference point, as estimated by linear regression adjusted for age, the presence of asthma, and contact lens usage.

	TIMP-1 (ng/mL)						
MMP-13 (ng/mL)		12.18	20.09	33.12	54.60	90.02	148.41
	33.12	*p* > 0.05	*p* > 0.05	*p* > 0.05	*p* > 0.05	*p* > 0.05	Reference point
	54.60	4.96 [0.27; 9.64] *p* = 0.038	*p* > 0.05	*p* > 0.05	*p* > 0.05	*p* > 0.05	*p* > 0.05
	90.02	4.30 [0.021; 8.59] *p* = 0.049	*p* > 0.05	*p* > 0.05	*p* > 0.05	*p* > 0.05	*p* > 0.05
	148.41	N/A	N/A	*p* > 0.05	*p* > 0.05	*p* > 0.05	*p* > 0.05
	244.69	N/A	N/A	*p* > 0.05	3.93 [0.084; 7.79] *p* = 0.045	4.60 [0.69; 8.51] *p* = 0.022	4.43 [0.29; 8.58] *p* = 0.037

Square brackets indicate 95% confidence intervals; N/A indicates insufficient sample coverage for estimation.

**Table 4:** Significant associations between pairs of mediators and the Standardized Pentacam Score.

	IL-6 (pg/mL)	CCL5 (pg/mL)	TIMP-1 (ng/mL)	t-PA (pg/mL)
CXCL8 (pg/mL)	*p* = 0.014	*p* = 0.028		*p* = 0.024
CCL5 (pg/mL)				*p* = 0.026
MMP-9 (ng/mL)		*p* = 0.04	*p* = 0.001	
MMP-13 (ng/mL)			*p* = 0.043	
t-PA (pg/mL)			*p* = 0.014	
PAI-1 (ng/mL)				*p* = 0.02

IL: interleukin; CXCL8: chemokine (C-X-C motif) ligand 8 = IL-8; CCL5: chemokine (C-C motif) ligand 5 = RANTES: regulated on activation, normal T cell expressed and secreted; MMP: matrix metalloproteinase; TIMP-1: tissue inhibitor of metalloproteinase-1; t-PA: tissue plasminogen activator; PAI-1: plasminogen activator inhibitor.
